# Substantial gap in primary care: older adults with HIV presenting late to care

**DOI:** 10.1186/s12877-020-01842-y

**Published:** 2020-10-31

**Authors:** Faiza Yasin, Christina Rizk, Bennie Taylor, Lydia A. Barakat

**Affiliations:** 1 Department of Medicine, Section of Infectious Diseases, Yale AIDS Program, Yale University School of Medicine, 135 College Street, Suite 323, New Haven, CT 06510 USA; 2 Yale AIDS Program, Yale University School of Medicine, New Haven, CT USA; 3 Whitman Walker Health, Washington, D.C, USA

**Keywords:** Aging population, Older adults, HIV, Risk, Testing, Stigma

## Abstract

**Background:**

Late diagnosis of human immunodeficiency virus (HIV) is associated with increased morbidity and mortality, and represents a serious public health concern.

**Methods:**

A retrospective medical record review was conducted on 188 patients with newly diagnosed HIV at a large academic center’s HIV clinic from 1/2010 to 12/2019. Patient demographic data, HIV staging, and response to combination antiretroviral therapy (cART) as measured by HIV viral suppression at 12 weeks (HIV RNA < 50 copies) were collected. Bivariate analyses were applied to compare patients ≥50 years old to those < 50 years old.

**Results:**

Over two-thirds of the older patients with a new diagnosis of HIV presented with a CD4 count < 200, or an AIDS-defining illness. Though not statistically significant, this same group also had a delay to viral suppression with only 59% achieving viral suppression after 12-weeks of cART initiation.

**Conclusions:**

This study suggests that older patients are presenting to care with advanced stages of HIV, and may also have a delay in achieving viral suppression after cART initiation. Future studies should aim to target HIV testing and treatment strategies for this at-risk older adult group.

## Background

Late testing and diagnosis remains prevalent in the United States (US) despite efforts to implement universal routine human immunodeficiency virus (HIV) testing guidelines for all adults in the US aged 13–65 [1, 2]. The definition of late diagnosis of HIV varies across the literature, but it generally describes people with HIV (PWH) who meet the Acquired Immunodeficiency Syndrome (AIDS) case definition within 12 months of their initial HIV diagnosis [3]. A diagnosis of HIV at a later stage is associated with increased morbidity and mortality [4, 5], risk of further HIV transmission [4, 6], increased healthcare costs, and decreased virologic and immunologic response to antiretroviral therapy [7, 8]. There are approximately 40,000 new HIV diagnoses annually in the United States [9], with an estimated 32–38% of whom develop AIDS within 12 months of their HIV diagnosis [10]. One in six of these newly diagnosed individuals is 50 years of age or older, and 35% of individuals in this age sub-group are diagnosed with late stage disease [3]. Some studies have found that older patients are often perceived to have lower risk for acquiring HIV, which is subsequently associated with a higher threshold for considering HIV on the differential for a presenting clinical syndrome that in a patient perceived at higher risk for HIV acquisition would prompt HIV testing [11, 12]. Furthermore, older patients are also often not included in the target population for larger scale routine HIV testing programs [12].

New Haven has one of the highest HIV prevalence rates in Connecticut, estimated by census data to be 1000/100,000 [3]. In 2018, the Connecticut Department of Public Health documented 258 new HIV cases, of which 59 (22.8%) were among individuals 50 years of age and older [3]. Of these new infections among older adults, 25 (42.0%) progressed to AIDS within 12 months of diagnosis [3]. The primary risk factor for HIV transmission among all new infections in Connecticut was male-to-male sexual contact, accounting for over 50% of new infections [3]. Based on 2018 prevalence data for individuals 50 years of age and older living with HIV in Connecticut, the primary risk factor in this subgroup is men who have sex with men (MSM) (30.9%), followed by high-risk heterosexual contact (27.7%), and then intravenous drug use (26.4%) [3].These findings suggest that older individuals make up a substantial portion of incident HIV infections in New Haven, and have a high risk of progressing to AIDS, raising the concern that there are barriers to testing, retention of care, and treatment for older adults living with HIV.

In order to better understand the characteristics and risk factors of older adults presenting late to care, in a region with a relatively high prevalence of HIV infection, we designed a study to evaluate the characteristics of new HIV infections diagnosed at an urban HIV clinic in New Haven, Connecticut.

## Methods

A retrospective medical record review of all patients newly diagnosed with HIV who initially presented for care was conducted at a single academic HIV clinic in New Haven, CT from 1/1/2010 to 12/31/2019. Institutional Human Investigation Committee approval was obtained.

Patient sex, race, risk factor for HIV acquisition, age group, insurance status, and HIV staging data were abstracted. In addition, the clinical response to combined antiretroviral treatment (cART) as measured by HIV viral suppression at 12 weeks (defined as HIV RNA < 50 copies/ml), and change in CD4 count were collected. Patients were categorized by age group, with the older population defined as patients who were 50 years of age or older at time of diagnosis, and the younger population defined as individuals less than 50 years of age at time of diagnosis.

Bivariate analyses using two-tailed t-test and Pearson’s chi-square test were used to compare characteristics of younger adults diagnosed with HIV to older adults diagnosed with HIV. The primary measures evaluated were an initial CD4 count < 200 or an AIDS-defining illness, based on Centers for Disease Control (CDC) criteria, at time of diagnosis, viral suppression, and change in CD4 count after 12 weeks of cART initiation. All statistical analyses were performed using R-studio version 1.1.456, and a *p*-value at the level of 0.05 was used to determine statistical significance.

A secondary review was conducted to assess for trends among the population of older adults diagnosed with HIV. This review focused on 8 themes which were identified a priori as risk modifiers for late HIV diagnosis, challenges with treatment adherence, and engagement in care: housing insecurity, substance use disorder, mental health, employment, sex, sexual practices, insurance status, and known history of prior HIV testing. Two independent reviewers coded the data in binary format, and identified key phrases from patient chart abstraction.

## Results

**Fig. 1:**
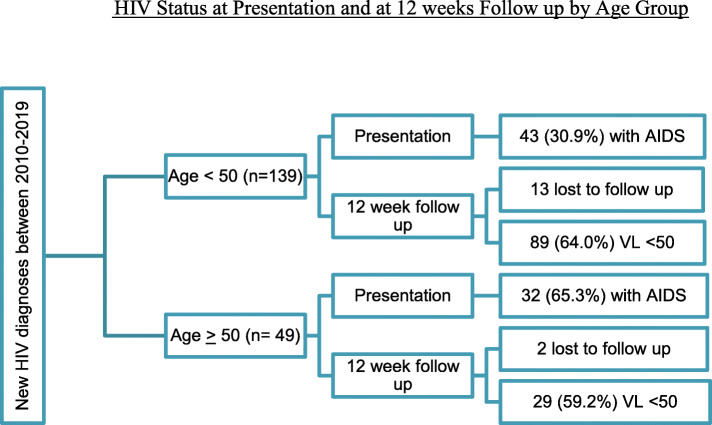
HIV Status at Presentation and at 12 weeks Follow up by Age Group

From 1/1/2010 to 12/31/2019, 188 patients met eligibility criteria and were included in the study. Of those, 139 patients (73.9%) were included in the younger patient group, categorized as less than 50 years of age, and 49 (26.1%) were included in the older patient group, categorized as 50 years of age or older (Fig. 1).

The majority of the younger group (*n* = 94, 67.6%) were non-white, compared to the older group who were primarily white (*n* = 26, 53.1%). Risk factors for HIV acquisition were similar across the two age groups, with 62.5% of the younger patients and 42.8% of the older patients reporting sexual activity with men, and identifying themselves as men who have sex with men. Most of the study participants (*n* = 176, 93.6%) were started on a cART regimen, primarily with an integrase strand transfer inhibitor (INSTI) (57.2%) or non-nucleoside reverse transcriptase inhibitor (NNRTI) (28.7%) anchor. Of the remaining 12 patients who were not started on cART, 7 were lost to follow up, 3 declined to start anti-retroviral agents, and 2 were not started on anti-retroviral agents because they were considered elite controllers (Fig. 1).

The majority of older patients (*n* = 32, 65.3%) presented with a CD4 count less than 200 cells/dl, or an AIDS defining illness compared to their younger counterparts (*n* = 43, 30.9%), which was a statistically significant finding (*p* < 0.0001) (Table 1). Mean CD4 count at time of diagnosis was 263.3 cells/dl, and significantly lower among the older age group compared to the younger group (*p* = 0.01), however, viral load at time of diagnosis was not significantly different (*p* = 0.97). Although the older group was more likely to have a delay in viral suppression at 12-weeks, with only 59.2% in the older group achieving viral suppression compared to 64.0% in the younger group, it was not statistically significant (*p* = N.S.). In addition, the older group had a smaller increase in CD4 count at 12 weeks compared to the younger group (average change in CD4 count 130 vs. 167 cells/dl respectively, *p* = N.S).

**Table 1:** Patients' characteristics by age group

Patients’ Characteristics by Age Group ***n*** = 188			
	Age < 50 *n* (%)	Age **>** 50 *n* (%)	*p*-value
Characteristic	139 (73.9%)	49 (26.1%)	
Sex			
Male	103 (74.1%)	38 (77.5%)	0.63
Female	36 (25.8%)	11 (22.4%)	
Race			
White	45 (32.4%)	26 (53.1%)	**0.01**
Non-White	94 (67.6%)	23 (46.9%)	
Risk Factor			
Heterosexual	39 (28.0%)	16 (32.6%)	0.16
MSM	87 (62.5%)	21 (42.8%)	
IVDU	1 (0.7%)	4 (8.2%)	
IVDU + MSM	2 (0.7%)	1 (2.0%)	
Unknown	10 (7.3%)	7 (14.3%)	
Insurance			
Uninsured	17 (12.2%)	0 (0%)	**0.03**
Insured	122 (87.8%)	49 (100.0%)	
*Private*	*62 (50.8%)*	*25 (51.0%)*	
*Public*	*59 (49.2%)*	*24 (49.0%)*	
cART class	127 (91.4%)	49 (100%)	
INSTI	75 (53.9%)	28 (57.1%)	0.29
NNRTI	43 (30.9%)	11 (22.4%)	
PI	7 (5.0%)	7 (14.3%)	
None	12 (8.6%)	0 (0%)	
Other	2 (1.4%)	3 (6.1%)	
Mean CD4 (cells/ml)	398.5	263.3	**0.01**
Mean HIV VL (copies/ml)	427,182.90	627,190.00	0.97
**AIDS diagnosis**	43 (30.9%)	32 (65.3%)	**0.00002**
**No AIDS diagnosis**	96 (69.0%)	17 (34.7%)	

Non-white = African American, Hispanic, Asian, Other

AIDS at diagnosis = CD4 < 200 or CDC AIDS defining illness

*MSM* Men who have Sex with Men, *IVDU* Injection Drug Use, *cART* Combination Antiretroviral Therapy, *INSTI* Integrase Strand Transfer Inhibitor, *NNRTI* Non-Nucleoside Reverse Transcriptase Inhibitor, *PI* Protease Inhibitor

Pearson’s chi-square test was used to analyze the following variables: race, sex. Two tailed t-test was used to analyze the following variables: ARV class, insurance, mean CD4 count, mean HIV viral load, and AIDS at diagnosis

### Content analysis

Additional data abstraction from chart review to elucidate common themes and structural barriers to care among those 50 years of age and older who presented with AIDS revealed substantial housing instability, mental health diagnoses, substance use disorder, and unemployment rates. Half (50.0%) of this group was identified by their health care provider as being both unemployed and also having housing insecurity within 1 year of their HIV diagnosis. Over one-third (30%) were identified as having a mental health disorder, most commonly depression and anxiety, and approximately one-third (31%) were identified as having a history of a substance use disorder. This data was limited by incomplete chart documentation, and lack of standardized documentation practices.

## Discussion

This study demonstrated that over two thirds of older adults have AIDS, as defined by CD4 count or AIDS defining illness, at the time of diagnosis, and that older patients may be less likely to achieve HIV virologic suppression at 12 weeks. These results are alarming and highlight several important findings.

Despite an overall decline in new HIV infections in all age categories in high resource countries, people 50 years of age and older are disproportionately affected by late HIV diagnosis [9]. Older patient populations may represent an at-risk group that is being missed in targeted comprehensive HIV prevention packages. According to a survey conducted by the Kaiser Family Foundation, as of 2014, only about half (54%) of U.S. adults aged 18–64 reported ever having been tested for HIV [13]. In our study, almost two-thirds (65.3%) of the older patient population with a new diagnosis of HIV at time of initial evaluation presented with AIDS, suggesting that early testing and treatment interventions are not reaching this population.

The majority of the data on risk factors for acquiring HIV and targeted testing strategies have focused on younger population demographics, and those thought to be at highest risk for acquiring HIV, such as individuals who use injection drugs and young MSM [12]. Older age groups have historically been considered to be “lower risk”, with some studies suggesting that older adults themselves do not perceive themselves to be at risk for HIV [14].

Our study suggests that testing based only on perceived risk may miss early detection of HIV among older adults, who then subsequently present with later stages of HIV at time of diagnosis. Furthermore, this group of patients experiences a delay in HIV diagnosis despite having contact with the healthcare system for other co-morbidities, and even in the presence of warning signs of HIV indicator conditions [15, 16]. Data review suggested approximately half of older participants were unemployed and experienced housing insecurity, and over one-third were diagnosed with a mental health disorder, suggesting that delay in diagnosis may be linked to lack of insurance or mental health illnesses. Furthermore, there is a major concern related to stigma associated with HIV that prevents physicians from offering HIV testing to their older patients [17, 18], and personal fear from stigma among patients themselves, which contributes to late diagnosis [19]. In particular, the literature highlights older MSM as being particularly vulnerable to stigma, in part based on MSM identity, but also in part due to increased susceptibility to social isolation and decreased social support with aging. This is similar to our study in which the majority (42.6%) of our older patients who were late to care were identified as MSM, though identifying social support systems was not a part of our data review. These findings emphasize the importance of universal HIV testing, and closing the gap on missed opportunities of early testing and linkage to care.

Late diagnosis of HIV is known to be associated with multiple serious adverse health outcomes, including increased morbidity and mortality to the individual due to the inflammatory process associated with HIV viremia. As described earlier, the older group of patients in our cohort presented with lower CD4 counts and a higher incidence of AIDS defining conditions compared to the younger group. This is a concerning finding with respect to overall morbidity and mortality, as the older group is also at a higher risk of cancer, cardiovascular disease, and neurocognitive impairment [20, 21]. In addition, a lower CD4 count is known to be associated with a higher risk of a new AIDS defining illness, and overall mortality, even after viral suppression on ART [22]. An important finding in our study was the delay in viral suppression after ART initiation in the older participants. Although not statistically significant, 40.8% of the older group did not achieve viral suppression at 12 weeks after cART initiation. This delay in viral suppression is associated with poor CD4 T-cell response and increases the individual and public health risks [23]. Overall, late diagnosis also poses a public health hazard by delaying entry to care, initiation of cART, and viral suppression, thereby increasing the potential risk of HIV transmission to partners.

This study had several limitations. Given the small cohort size from a single academic medical center in an urban setting, findings may not be generalizable to the larger nation-wide population. The modest sample size, notably within the older age group (*n* = 49), also did not allow sub-group analyses which limited the power of the study. However, our data was similar to already published data in larger cohorts, and affirmed the firm conclusions about themes and gaps in care identified. The data highlight several additional areas of future study, including more robust qualitative study on the risk factors identified in older patients living with HIV. It is also plausible that there is subgroup variability among the patients over 50 who are in the 50–65-year-old range compared to the older than 65-year-old group. Additional study with subgroup analysis is needed to evaluate the differences between these age groups, which may further inform HIV testing guidelines by age group. The retrospective nature of the study also limits qualitative analysis, as the a priori identified themes were not consistently documented in the electronic medical records. It highlights an area of research moving forward that would allow for directed services to persons living with HIV, with a focus on qualitative data to fully understand the intricacies of the themes that emerged in chart review. Finally, our data did not include information on the interval between time of HIV diagnosis to cART initiation, which is a limitation in our analysis.

## Conclusion

In conclusion, this study highlights that late diagnosis of HIV among older patients is an ongoing and inadequately targeted problem. A focus on identifying specific risk factors and unmet needs associated with older patients is urgently required. Our findings reaffirm the importance of implementing the CDC recommended routine HIV screening and targeted prevention methods to older adults as a necessary next step in primary care settings.

## Data Availability

The datasets used and analyzed during the current study are available from the corresponding author on reasonable request.

## Abbreviations

HIV: Human Immunodeficiency Virus
cART: Combination antiretroviral therapy
ART: Anti-retroviral therapy
US: United States
AIDS: Acquired Immunodeficiency Syndrome
MSM: Men who have sex with men
CDC: Centers for Disease Control
INSTI: Integrase strand transfer inhibitor
NNRTI: Non-nucleoside reverse transcriptase inhibitor
PI: Protease inhibitor
VL: Viral load
IVDU: Intravenous drug use
